# Developing regional genetic counseling for southern Chinese with nonsyndromic hearing impairment: a unique mutational spectrum

**DOI:** 10.1186/1479-5876-12-64

**Published:** 2014-03-11

**Authors:** Kaitian Chen, Ling Zong, Min Liu, Xianren Wang, Wei Zhou, Yuan Zhan, Hui Cao, Chang Dong, Haocheng Tang, Hongyan Jiang

**Affiliations:** 1 Department of Otorhinolaryngology, the First Affiliated Hospital, Sun Yat-sen University and Institute of Otorhinolaryngology, Sun Yat-sen University, Guangzhou 510080, People's Republic of China

**Keywords:** South China, Non-syndromic hearing impairment, *GJB2*, *SLC26A4*, Mitochondrial DNA, *GJB3*, *WFS1*, Gene mutation

## Abstract

**Background:**

Racial and regional factors are important for the clinical diagnosis of non-syndromic hearing impairment. Comprehensive genetic analysis of deaf patients in different regions of China must be performed to provide effective genetic counseling. To evaluate the mutational spectrum of south Chinese families, we performed genetic analysis for non-syndromic hearing impairment in this population.

**Methods:**

Complete clinical evaluations were performed on 701 unrelated patients with non-syndromic hearing impairment from six provinces in south China. Each subject was screened for common mutations, including *SLC26A4* c.IVS7-2A > G, c.2168A > G; mitochondrial DNA m.1555A > G, m.1494C > T, m.7444G > A, m.7445A > G; *GJB3* c.538C > T, c.547G > A; and *WFS1* c.1901A > C, using pyrosequencing. *GJB2* and *SLC26A4* coding region mutation detection were performed using Sanger sequencing.

**Results:**

Genetic analysis revealed that among the etiology of non-syndromic hearing impairment, *GJB2*, *SLC26A4*, and mitochondrial m.1555A > G mutations accounted for 18.0%, 13.1%, and 0.9%, respectively. Common mutations included *GJB2* c.235delC, c.109G > A, *SLC26A4* c.IVS7-2A > G, c.1229 T > C, and mitochondrial m.1555A > G. The total mutation rate was 45.1% in all patients examined in south China. Overall, the clear contribution of *GJB2*, *SLC26A4*, and mitochondrial m.1555A > G to the etiology of the non-syndromic deafness population in south China was 32.0%.

**Conclusions:**

Our study is the first genetic analysis of non-syndromic hearing impairment in south China, and revealed that a clear genetic etiology accounted for 32.0% of non-syndromic hearing cases in patients from these regions. The mutational spectrum of non-syndromic hearing impairment in the south Chinese population provides useful and targeted information to aid in genetic counseling.

## Background

Hereditary deafness is a genetically heterogeneous disease with an incidence rate of approximately 1/1000 [1]. Non-syndromic hearing impairment (NSHI) accounts for 60-70% of inherited hearing impairments. Among the plethora of deafness genes discovered in the past decade, certain genes are more important than others from an epidemiologic perspective. Mutations in *GJB2*, *SLC26A4*, and the mitochondrial 12SrRNA gene have been shown to have a high prevalence in NSHI [2-4].

China is a multiethnic, large country with a population of at least 1.6 billion, which encompasses 9.6 million square kilometers. Moreover, there are 27.80 million Chinese people with hearing and speech disabilities; of these, 20.04 million have a simple hearing disability [5]. Although a few genetic studies have been conducted on Chinese patients with deafness [5-8], the large deafness population and racial differences demand regional and individual genetic analysis; such data cannot be simply inferred from other groups’ conclusions. For example, in typical areas of China (Chifeng City in Inner Mongolia and Nangtong City in JiangSu Province), *GJB2* gene mutations account for the etiology of approximately 18.31% of patients with hearing loss, *SLC26A4* mutations account for approximately 13.73%, and the mitochondrial m.1555A > G mutation accounts for 1.76% [7]. However, common molecular etiologies are rare in non-syndromic Tibetan Chinese patients with hearing impairment [5]. No homozygous or compound heterozygous mutation of the *GJB2* or *SLC26A4* gene was found, and the mitochondrial m.1555A > G homogeneous mutation were responsible for hearing loss in 1.75% of 114 Tibetan patients.

**Figure 1:**
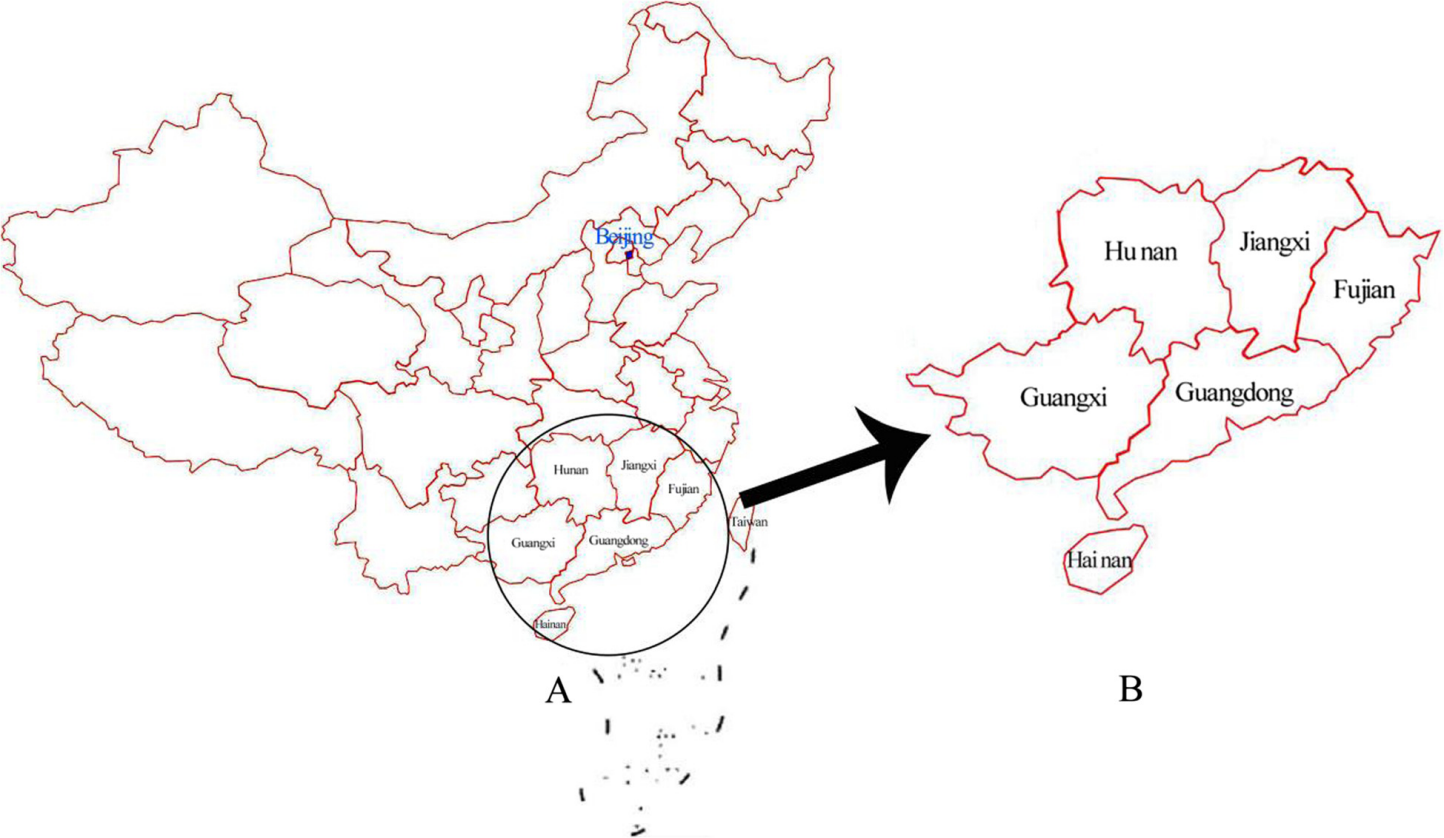
Geographical distribution of patients from six provinces (circle in A) and an enlarged partial view (B).

Genetic counseling for deafness should fully take into account the differences in regional backgrounds. Therefore, the establishment of a specific mutational database for different regional populations is indispensable. Although there are northern [9], central region [7], Xinjiang [10], Yunnan [11] and Tibet Chinese [5], the mutation spectrum of NSHI in patients from south China has not been examined (Figure 1). To extend the epidemiological data on common gene mutations in the Chinese population and to provide appropriate genetic testing and counseling for patients with NSHI in south China, we conducted a genetic analysis in this population.

## Methods

A total of 701 unrelated sporadic or familial cases of NSHI were recruited from six provinces in south China, including Guangdong, Guangxi, Hai’nan, Hu’nan, Fujian and Jiangxi (Figure 1). Each patient underwent a complete medical history and physical examination to exclude the possibility of non-genetic causes or any syndromic findings. An age-appropriate audiological examination was performed, including pure-tone audiometry (PTA) and/or auditory brainstem response (ABR), auditory steady state response (ASSR), immittance testing, and distortion product otoacoustic emissions (DPOAE). Temporal computed tomography (CT) examination was conducted in 664 patients. Among these patients, 134 patients were diagnosed as enlarged vestibular aqueduct (EVA) and 530 patients had normal CT appearance. The rest 37 patients or their guardians refused to receive temporal CT examination. For control purposes, the study included 180 age-matched individuals with normal hearing. The study was approved by the institutional review board of the First Affiliated Hospital, Sun Yat-sen University. Informed consent was obtained from all patients or their guardians.

Blood samples were collected from the subjects, and DNA isolation was performed using a standard chloroform extraction method. Targeted sequences were then amplified by PCR. The coding exon (Exon 2) and flanking intronic regions of *GJB2* gene were PCR amplified with forward primer 5′TTGGTGTTTGCTCAGGAAGA 3′ and reverse primer 5′GGCCTACAGGGGTTTCAAAT 3′ [8]. Patients with monoallelic *GJB2* coding region mutations were additionally tested for *GJB2* c.IVS1 + 1G > A mutation or defects in *GJB2* exon 1 and its basal promoter [11,12].

Pyrosequencing was then utilized to detect *SLC26A4* c.IVS7-2A > G, c.2168A > G; mitochondrial m.1555A > G, m.1494C > T, m.7444G > A, m.7445A > G; *GJB3* c.538C > T, c.547G > A, and *WFS1* c.1901A > C in each subject. The biotinylated PCR primers were designed according to the Pyrosequencing Assay Design Software (Qiagen, Hilden, Germany). Detailed procedures have been provided by the manufacturer and previous study [13]. Briefly, bound biotinylated single-stranded DNA was generated using PSQ 96 Sample Preparation Kit (Qiagen, Hilden, Germany). After template preparation for pyrosequencing, an automated Pyrosequencing instrument, PSQ96 (Qiagen, Hilden, Germany), was used to perform genotyping. The resulting sequences were analyzed manually by visual inspection of each pyrogram, automatically by the SNP evaluation software (Qiagen, Hilden, Germany), or by both approaches.

Furthermore, 21 exons and flanking sequences from the *SLC26A4* gene were directly sequenced using an ABI 3730 Genetic Sequencer as previous study [14] of patients with EVA. Sequences were aligned and compared with published sequences from the NCBI database (*GJB2*: NG_008358.1; *SLC26A4*: NG_008489.1).

## Results

### Geographical and clinical characteristics of patients

We enrolled 701 unrelated patients (417 males and 284 females) diagnosed with NSHI from south China. The geographical distribution covered six provinces (Figure 1). Specifically, there were 238 cases from Guangdong, 92 cases from Jiangxi, 98 cases from Guangxi, 96 cases from Fujian, 98 cases from Hainan, and 79 cases from Hu’nan. The average patient age was 7.5 years (range: 11 months-47 years), and 97.3% of the patients were Han Chinese. Each individual demonstrated congenital hearing loss or postlingual bilateral deafness that ranged from mild to anacusis. The average onset age of patients with postlingual NSHI was 8.71 ± 7.24 years. No goiter was discovered.

### Prevalent GJB2 mutations in patients with NSHI from south China

*GJB2* coding region sequencing identified ten known pathogenic mutations (c.11G > A, c.35delG, c.109G > A, c.176-191del16, c.235delC, c.257C > G, c.299-300delAT, c.427C > T, c.512insAACG, and c.605ins46), two unclassified variants (c.187G > T, c.368C > A), and five polymorphisms (c.79G > A, c.341A > G, c.186C > T, c.558G > A and c.608 T > C). All *GJB2* mutations identified in this cohort were recessive. Dominant mutation was absent in this cohort.

In total, 126 patients (18.0%, 126/701) carried a homozygous or compound heterozygous *GJB2* pathogenic mutation (Table 1). The mutation c.235delC is also the most prevalent among south Chinese; but c.109G > A is most frequent in south Chinese than in north Chinese. Another prevalent mutation, c.299-300delAT [4,8] was less common, displaying a discrepant genetic spectrum in south Chinese. As to allele frequency, the c.235delC mutation was most commonly observed (46.8%, 118/252), followed by the c.109G > A (40.1%, 101/252) and c.299-300delAT mutations (4.76%, 12/252).

**Table 1:** ***GJB2 *** **mutation spectrum in patients with NSHI from south China**

**Allele 1**		**Allele 2**		**No. (Proportion)**
**Nucleotide**	**AA change**	**Nucleotide**	**AA change**	
c.109G > A	p.V37I	c.109G > A	p.V37I	44(34.9%)
c.235delC	Frameshift	c.235delC	Frameshift	42(33.3%)
c.235delC	Frameshift	c.109G > A	p.V37I	12(9.52%)
c.235delC	Frameshift	c.299-300delAT	Frameshift	9(7.1%)
c.235delC	Frameshift	c.176-191del16	Frameshift	5(4.0%)
c.235delC	Frameshift	c.512insAACG	Frameshift	4(3.17%)
c.235delC	Frameshift	c.35delG	Frameshift	1(0.8%)
c.235delC	Frameshift	c.257C > G	p.T86R	1(0.8%)
c.299-300delAT	Frameshift	c.299-300delAT	Frameshift	1(0.8%)
c.512insAACG	Frameshift	c.257C > G	p.T86R	2(1.6%)
c.235delC	Frameshift	c.427C > T	p.R143W	1(0.8%)
c.235delC	Frameshift	c.605ins46	Frameshift	1(0.8%)
c.257C > G	p.T86R	c.176-191del16	Frameshift	2(1.6%)
c.109G > A	p.V37I	c.299-300delAT	Frameshift	1(0.8%)

*GJB2* mono-allelic mutations are shown in Table 2. These patients with were additionally tested for *GJB2* c.IVS1 + 1G > A mutation or defects in *GJB2* exon 1 and its basal promoter, however, no pathogenic mutation was identified. The proportion of c.109G > A heterozygosity was 10.7% in patients with NSHI compared to approximately 6.1% in 180 normal control samples. *GJB3* or *GJB6* coding region mutations were further examined in some of these patients and no pathogenic mutation was detected [15].

**Table 2:** **Summary of *GJB2 *** **mono-allelic mutations in patients with NSHI from south China**

**Nucleotide**	**AA change**	**Category**	**No. (Proportion)**
c.109G > A	p.V37I	Pathogenic	75(10.7%)
c.235delC	Frameshift	Pathogenic	5(0.71%)
c.512insAACG	Frameshift	Pathogenic	3(0.42%)
c.187G > T	p.V63L	Unclassified	2(0.29%)
c.186C > T	p.N62N	Polymorphism	1(0.14%)
c.558G > A	p.T186T	Polymorphism	2(0.29%)
c.11G > A	p.G4D	Pathogenic	2(0.29%)
c.368C > A	p.T123N	Unclassified	2(0.29%)
c.608 T > C	p.I203T	Polymorphism	6(0.85%)

### Hereditary heterogeneity of SLC26A4 mutations correlated with EVA

We identified 80 patients from 134 patients with EVA carrying at least one *SLC26A4* c.IVS7-2A > G or c.2168A > G allele by pyrosequencing. In contrast, five c.IVS7-2A > G and three c.2168A > G heterozygous alleles were detected in 530 NSHI patients with normal temporal CT appearance. Another 37 patients without temporal CT examination were negative for c.IVS7-2A > G or c.2168A > G mutation. These results were confirmed by Sanger sequencing. *SLC26A4* exons sequencing further confirmed that 92 patients carried biallelic mutations and five patients exhibited mono-allelic mutations in the 134 patients with EVA. *SLC26A4* accounts for approximately 13.1% (92/701) of the cases of NSHI in this population (Table 3), secondary to the contribution of *GJB2*.

**Table 3:** ***SLC26A4 *** **mutation spectrum in patients with EVA from south China**

**Allele 1**		**Allele 2**		**No.**
**Nucleotide**	**AA change**	**Nucleotide**	**AA change**	
c.IVS7-2A > G	Splice site	c.IVS7-2A > G	Splice site	29
c.IVS7-2A > G	Splice site	c.2086C > T	p.Q696Stop	5
c.IVS7-2A > G	Splice site	c.2168A > G	p.H723R	5
c.IVS7-2A > G	Splice site	c.1229C > T	p.T410M	9
c.IVS7-2A > G	Splice site	c.2000 T > C	p.F667S	1
c.IVS7-2A > G	Splice site	c.1691insA	Frameshift	3
c.IVS7-2A > G	Splice site	c.1343C > T	p.S448L	3
c.IVS7-2A > G	Splice site	c.754 T > C	p.S252P	4
c.IVS7-2A > G	Splice site	c.1369A > T	p.N457Y	1
c.IVS7-2A > G	Splice site	c.1079C > T	p.A360V	4
c.IVS7-2A > G	Splice site	c.1540C > T	p.Q514Stop	2
c.IVS7-2A > G	Splice site	c.1919G > A	p.W641Stop	2
c.IVS7-2A > G	Splice site	c.IVS14-2A > G	Splice site	1
c.IVS7-2A > G	Splice site	c.422 T > C	p.F141S	1
c.IVS7-2A > G	Splice site	c.259G > T	p.D87Y	1
c.754 T > C	p.S252P	c.754 T > C	p.S252P	1
c.754 T > C	p.S252P	c.2168A > G	p.H723R	1
c.754 T > C	p.S252P	c.1229C > T	p.T410M	1
c.1229C > T	p.T410M	c.1229C > T	p.T410M	1
c.1229C > T	p.T410M	c.1547insC	Frameshift	3
c.1229C > T	p.T410M	c.259G > T	p.D87Y	3
c.1229C > T	p.T410M	c.2086C > T	p.Q696Stop	2
c.1229C > T	p.T410M	c.679G > C	p.A227P	1
c.589G > A	p.G197R	c.1547insC	Frameshift	1
c.2086C > T	p.Q696Stop	c.2086C > T	p.Q696Stop	1
c.2168A > G	p.H723R	c.2168A > G	p.H723R	3
c.2168A > G	p.H723R	c.1975G > C	p.V659L	1
c.754 T > C	p.S252P	c.1975G > C	p.V659L	1
c.1975G > C	p.V659L	c.2086C > T	p.Q696Stop	1

*SLC26A4* exon sequencing confirmed four polymorphisms (c.IVS10 + 27C > A, c.IVS11 + 47 T > C, c.IVS19-24 T > A, and c.IVS17-74G > A); 20 probable pathogenic mutations, including 18 known mutations (c.259G > T, c.413 T > A, c.589G > A, c.679G > C, c.754 T > C, c.IVS7-2A > G, c.1079C > T, c.1229C > T, c.1343C > T, c.1540C > T, c.1547insC, c.IVS14-2A > G, c.1691insA, c.1919G > A, c.1975G > C, c.2000 T > C, c.2086C > T and c.2168A > G) and two novel mutations (c.422 T > C, c.1369A > T). We identified that the c.IVS7-2A > G mutation was most prevalent (54.5%, 103/189), followed by c.1229C > T (11.1%, 21/189, Table 3).

### Low frequency of mitochondrial DNA 1555A > G mutation

**Figure 2:**
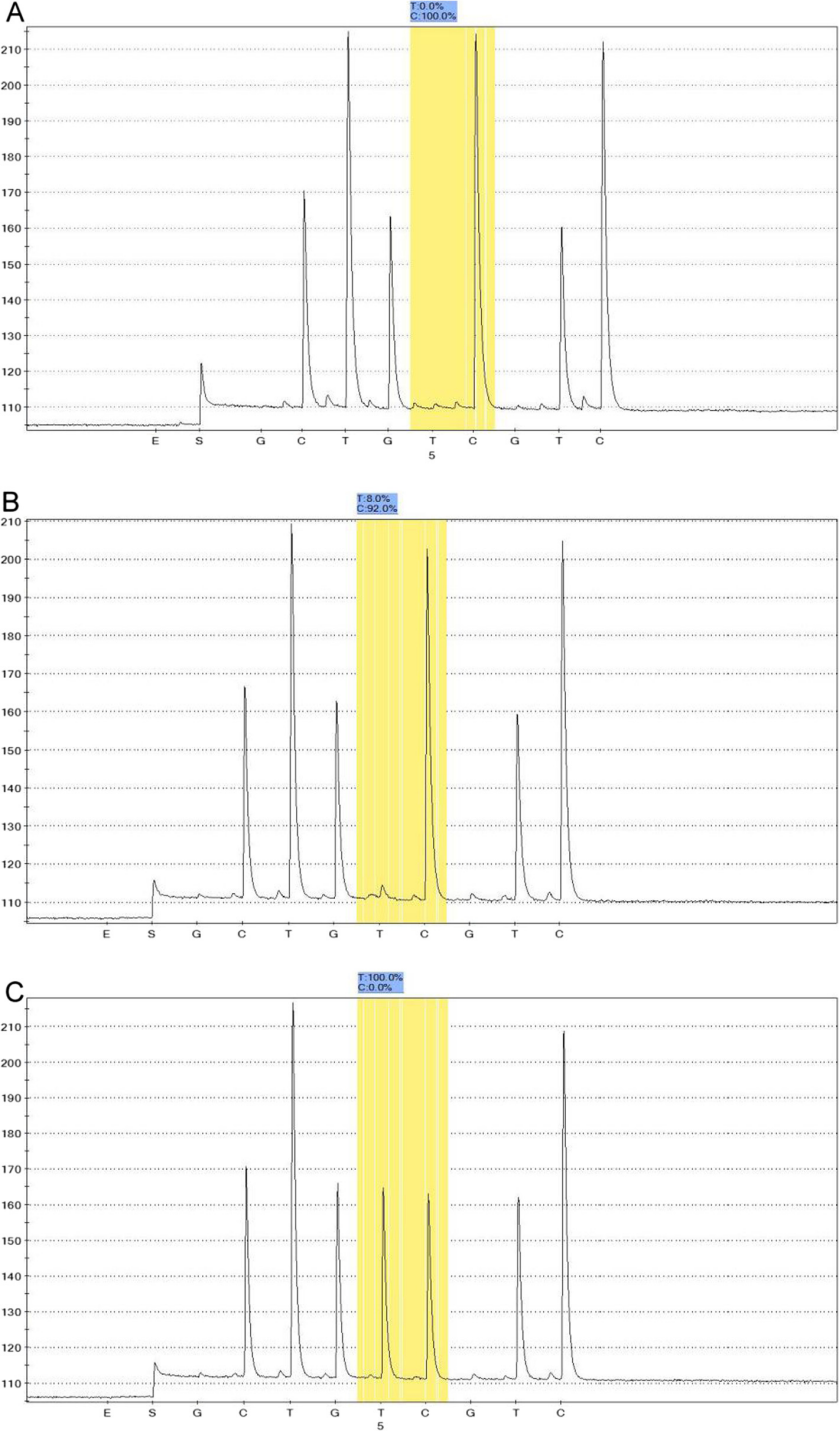
Pyrosequencing detection of mitochondrial DNA m.1555A > G homoplasmy (A), heteroplasmy (B), and wild-type (C).

Pyrosequencing identified an m.1555A > G mutation in the mitochondrial DNA of six patients (Figure 2), and four homoplasmy and two heteroplasmy mutations (the mutational threshold was 92% and 96%, respectively). In addition, a history of aminoglycoside exposure before the onset of deafness was confirmed in these six cases. Additional mutations, such as m.1494C > T, tRNAser^(UCN)^ m.7444G > A, and m.7445A > G, were absent. Therefore, we failed to locate a common mitochondrial DNA mutation in this cohort (0.9%, 6/701).

### GJB3 and WFS1 gene mutations are rare

In total, 701 patients lacked *GJB3* c.538C > T, c.547G > A, and *WFS1* c.1901A > C mutations, suggesting that these variants are rare in south Chinese. Moreover, 200 individuals were further examined for *GJB3* and *GJB6* coding region mutations, including del(*GJB6*-D13S1830) and del(*GJB6*-D13S1854) sequences; however, only two subjects were heterozygous for c.250G > A or c.580G > A mutations [15].

### Genetic counseling for south Chinese patients with NSHI

**Figure 3:**
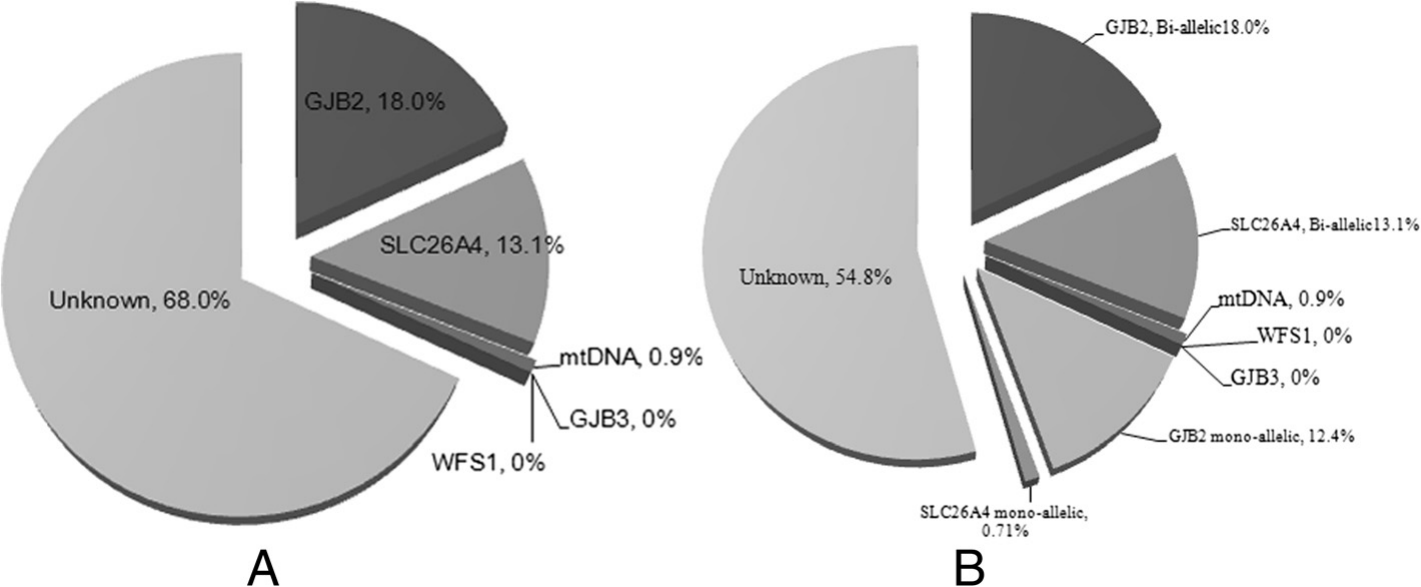
**The significance of genetic counseling services for NSHI in the south Chinese population. (A)** In total, 32% of the south China population of patients with NSHI could be diagnosed with hereditary deafness. **(B)** The carrying frequency of a common mutation was 45.1% in the south China population of patients with NSHI.

The total carrier mutation rate constituted approximately 45.1% in our patient cohort, with a clear genetic etiology in 32.0% of the patients with NSHI (Figure 3).

## Discussion

Based on a relatively large cohort, the present study analyzed the mutational spectrum of NSHI in the population of south China for the first time. It has been established that the common deafness mutations for the Chinese population are *GJB2* c.235delC, c.299-300delAT, c.176-191del16, c.35delG; *GJB3* c.538C > T; *SLC26A4* c.IVS7-2A > G, c.2168A > G; and mitochondrial m.1555A > G and m.1494C > T [8,10], whereas the major mutations in the south Chinese population consist of *GJB2* c.235delC, c.109G > A, c.299-300delAT, c.176-191del16; *SLC26A4* c.IVS7-2A > G and c.1229 T > C; and mitochondrial m.1555A > G. *GJB2* exon sequencing is recommended for each individual because it is simple, cheap (25 RMB per sample), and can provide more information (18%) than gene chips [10]. *SLC26A4* exons sequencing is suggested for patients with EVA. An extended and systematic study of the complete mitochondrial DNA sequence may provide significant further information for assessment of patients with NSHI.

China consists of 56 ethnic countries that comprise a population of over 1.6 billion. Given the heterogeneity of genetic deafness, ethnic and regional specific factors are important. Although genetic analysis of patients from the northern [9], central [7], Xinjiang [10], Yunnan [11], and Tibet [5] areas of China have been reported, the mutational spectrum of NSHI in south China - consisting of the Guangdong province and the surrounding areas - remained unclear. A genetic study of the south China population may provide regional-specific mutational information to improve the efficacy of clinical genetic counseling. Therefore, the present results provide useful information for clinical application of mutant gene detection in patients with deafness.

*GJB2* gene mutations are diverse in different ethnic regions (between 10–48.1%). The hereditary deafness website (http://hereditaryhearingloss.org) lists at least 111 *GJB2* mutations. The c.35delG mutation is most prevalent in Europe and the United States, compared with c.235delC in East Asian populations and c.167delT in Jewish populations [16]. We observed a *GJB2* mutation distribution of approximately 20% that was responsible for NSHI in south China. The *GJB2* mutation rate in south China was identical to that of central and northern China [4,6-11]. The c.109G > A mutation was very common, although its pathogenicity remains controversial. A study indicated that the c.109G > A mutation had an allelic frequency of 6.7% (185/2744) in a Han patient group (excluding all cases with two clearly pathogenic mutations); this rate was significantly higher than the control population (2.8%, 17/602; *P* =0.0003) [17]. These results support the conclusion that *GJB2* c.109G > A should be reassigned to a pathogenic mutation. Our cell-based in vitro functional assays study also indicated that c.109G > A homozygote could impair connexin26 gap junctional coupling and should be assigned to pathogenic mutation (unpublished data). In addition, there is a lack of genetic information for the *GJB2* mono-allelic mutation, which makes genetic counseling for these patients more difficult. The frequency of c.109G > A in control group is relatively high (6.1%) in our cohort. This may partially be attributed to the small sample sizes and/or selection bias; However, this frequency might reflect the prevalence of c.109G > A in this cohort and c.109G > A might be ascribed to founder effect in south Chinese. Further population-based and functional studies are recommended.

*SLC26A4* mutations in Western countries and Asian populations show regional and ethnic diversity in their frequency and display mutational hot spots. For example, *SLC26A4* mutations account for approximately 4% of NSHI in the Caucasian population [18]. Evaluation of previous Chinese domestic studies indicates that the incidence of the *SLC26A4* mutation may be between 12-14% in patients with NSHI [19,20]. Our finding (13.1%) is consistent with these results, illuminating the feasibility of the *SLC26A4* detection strategy inferred from north China [7,9]. The c.IVS7-2A > G mutation is common in Eastern Asia. Several large cohort investigations have reported a high prevalence of c.IVS7-2A > G in the Chinese population (22.3-64.63%). In Koreans, the c.IVS7-2A > G mutation accounts for 20% of mutant *SLC26A4* alleles and is the second most prevalent genetic variant [21]. The c.IVS7-2A > G mutation has a low incidence of approximately 3% in Japanese individuals [22]. The distribution of mutant *SLC26A4* alleles revealed by our study suggests that mutation screening of four exons (exons 19, 10, 17 and 15) following c.IVS7-2A > G identification should be a priority for NSHI genetic testing in patients with EVA. Besides, previous studies [7,10,11,14,18] have revealed the close relationship between *SLC26A4* mutations and EVA. The absence of *SLC26A4* mutation in 37 patients without temporal CT in our cohort could preliminarily exclude the possibility of EVA in these patients.

Mitochondrial m.1555A > G mutation was detected at a lower rate in southern China compared with previous reports. The m.1555A > G mutation is observed in approximately 0.6-2.5% of Caucasian patients with NSHI [23], but more frequently in the Asian population, including China 2.9%, Japan 3% [24], and Indonesia 5.3% [25]. The tRNAser^(UCN)^ m.7445A > G and m.7444G > A mitochondrial mutations may be involved in clinical phenotype modifications. However, we failed to detect tRNAser^(UCN)^ m.7445A > G and m.7444G > A mutations; therefore, they may be rare in the south China population.

Previous studies have found that digenic inheritance of NSHI is caused by mutations in the *GJB2* and *GJB3* genes [26]. However, we observed that both *GJB3* and *GJB6* exhibited a low mutation rate [27,28]. In the present study, 701 cases lacked the c.538C > T or c.547G > A mutations in *GJB3*. Additional screening of 200 subjects for mutations in the *GJB3* and *GJB6* coding regions did not identify any pathogenic mutations [15]. Meanwhile, lack of *WFS1* c.1901A > C, a rare low-frequency mutation [29], excludes the necessity of routine screening for this allele. Therefore, our preliminary results indicate that the *GJB3, GJB6,* and *WFS1* c.1901A > C common gene mutations may be minor in NSHI and are not recommended as alleles for first-line screening.

Individualized genetic screening in south China may improve the NSHI diagnostic efficiency and positivity rate, to avoid unnecessary waste of economic and human resources. Therefore, larger epidemiological studies of the genetic etiology of NSHI are recommended due to the sample size limitations of the present study. New genetic technology, such as targeted exon sequencing, may shed further light on the genetic components of NSHI in patients that lack common gene mutations.

## Conclusions

We have conducted the first genetic analysis of NSHI in south China, and revealed that a clear genetic etiology accounted for 32.0% of cases. The NSHI mutational spectrum of the south Chinese population provides useful and important information for genetic counseling.

## Competing interests

The authors declare that they have no competing interests.

## Authors’ contributions

KC designed the study, collected clinical data, carried out genetic studies, participated in the sequence alignment and drafted the manuscript. LZ, ML, XW, WZ collected clinical data, carried out genetic studies, participated in the sequence alignment and revised the manuscript. YZ, HC, CD and HT collected clinical data, performed statistical analysis and wrote the manuscript. HJ conceived of the study, and participated in its design and coordination and manuscript revising. All authors read and approved the final manuscript.
